# Effect of chronic stress on running wheel activity in mice

**DOI:** 10.1371/journal.pone.0184829

**Published:** 2017-09-19

**Authors:** Evan DeVallance, Dale Riggs, Barbara Jackson, Travis Parkulo, Stanley Zaslau, Paul D. Chantler, I. Mark Olfert, Randy W. Bryner

**Affiliations:** 1 Division of Exercise Physiology, WVU School of Medicine, Morgantown, West Virginia, United States of America; 2 Department of Surgery, WVU School of Medicine, Morgantown, West Virginia, United States of America; Technion Israel Institute of Technology, ISRAEL

## Abstract

Acute and chronic stress have been reported to have differing effects on physical activity in rodents, but no study has examined a chronic stress protocol that incorporates stressors often experienced by rodents throughout a day. To examine this, the effects of the Unpredictable Chronic Mild Stress (UCMS) protocol on voluntary running wheel activity at multiple time points, and/or in response to acute removal of chronic stress was determined. Twenty male Balb/c mice were given access and accustomed to running wheels for 4 weeks, after which they were randomized into 2 groups; exercise (EX, n = 10) and exercise with chronic stress using a modified UCMS protocol for 7 hours/day (8:00 a.m.-3:00p.m.), 5 days/week for 8 weeks (EXS, n = 10). All mice were given access to running wheels from approximately 3:30 p.m. to 7:30 a.m. during the weekday, however during weekends mice had full-time access to running wheels (a time period of no stress for the EXS group). Daily wheel running distance and time were recorded. The average running distance, running time, and work each weekday was significantly lower in EXS compared to EX mice, however, the largest effect was seen during week one. Voluntary wheel running deceased in all mice with increasing age; the pattern of decline appeared to be similar between groups. During the weekend (when no stress was applied), EXS maintained higher distance compared to EX, as well as higher daily distance, time, and work compared to their weekday values. These results indicate that mild chronic stress reduces total spontaneous wheel running in mice during the first week of the daily stress induction and maintains this reduced level for up to 8 consecutive weeks. However, following five days of UCMS, voluntary running wheel activity rebounds within 2–3 days.

## Introduction

Acute stress may exert both positive and negative physiological responses, but chronic stress is known to result in many pathological conditions, including depression and other altered behaviors [1–3]. For example, chronic stress is often associated with an exaggerated activation of the hypothalamic-pituitary-adrenal (HPA) axis and elevated glucocorticoid levels [4], both of which have been linked to anxiety and affective disorders [5]. It has recently been reported that mice bred for high voluntary wheel running have been reported to have an enhanced HPA activation as indicated by elevated glucocorticoid levels [6]. The investigators attributed these latter responses to the chronically high glucocorticoid levels, which could be an evolutionary response to the greater need for energy with high aerobic capacity. It was also reported in these mice that acute stress (i.e. running wheel deprivation) resulted in an increased depressive-like behavior defined as reduced swimming activity when compared with control animals [7]. It could therefore be hypothesized that strains of mice not bred for robust wheel running activity may display similar depressive-like behavior including a reduced willingness to run when exposed to chronic stress. This becomes especially important if mice are continuously exposed to these types of stressors in their normal environment.

The Unpredictable Chronic Mild Stress (UCMS) protocol has been used for over two decades in neural and behavioral experiments to elicit depressive-like behaviors in rodents [8,9]. It consists of several stressors that the animal is exposed to in a random and ever changing fashion which prevents habituation. This protocol has been shown to cause increased levels of corticotrophin releasing factor (CRF), increased circulating corticosterone and glucose intolerance [10], and mirror neurobiological changes often observed in depressive disorders seen in clinical populations [11]. It has also been shown to cause anhedonia in rodents as evident by the fact that they have decreased desire for sucrose-sweetened drinks and other natural rewards [8,12]. Although meant to be a continuous mild stress stimulus at a level much greater than would normally be experienced by a rodent, the UCMS protocol does employ various stressors, some of which may be experienced by rodents in their normal daily environment (i.e., wet or limited bedding, changing of cages, unwanted social interactions, lights turned on during the dark cycle).

Acute stresses have been reported to increase the short term running activity in rodents during or immediately following the stress [13,14]. However, studies examining the long-term application of daily stressors on voluntary wheel running activity have not been conducted. It remains unclear whether exercise behavior changes between acute versus chronic stress, and whether the changes are incremental in nature or represent discrete step-wise changes. To our knowledge, no studies thus far have examined the effects of UCMS on the acute versus chronic voluntary running behavior in rodents. Voluntary wheel running, unlike forced treadmill running [15,16], allows the animal to freely exercise with minimal or no external stress [17]. Nevertheless, daily handling and other environmental stressors may themselves effect voluntary exercise behavior activity. Because of the increasing popularity of using voluntary wheel running in biomedical research, it is important to fully understand the effect of chronic stress on wheeling running behavior.

The purpose of this study was to evaluate the acute effect of 5 days of stress using the UCMS protocol for 7 hours each day on voluntary overnight wheel running activity. We also evaluated the running behavior during weekends when no UCMS was given. Finally, we determined the long-term effect of daily weekday-stress on running wheel activity by examining running behavior to a modified-UCMS protocol for an 8 week period. This design was used so that we could determine if a mild chronic stress applied most days of the week would cause progressive deterioration in running behavior over time or if the negative effects were more related to the acute effect of each days stress load. We hypothesized that the daily stress would reduce overnight wheel running in mice and that this reduction would become greater over the course of the 8-week study.

## Materials and methods

*Animals*: Eight-week-old male Balb/c mice (n = 20) were purchased from Harlan Laboratories (Indianapolis, IN) and used in this study. Balb/c mice were chosen because they have previously been shown to be susceptible to the UCMS protocol [18,19]. Mice were housed individually at the West Virginia University (WVU) Robert C. Byrd Health Science Center animal vivarium. The housing quarters are regulated at a constant temperature and on a 12-hour light: dark cycle (Lights on at 6:30 a.m., off at 6:30 p.m.). Mice were provided tap water and normal chow diet (Teklad Diet; 18% fat, 24% protein, 58% carbohydrates) *ad libitum*, and cages and bedding were changed at least once per week. This study was approved by the West Virginia University Institutional Animal Care and Use Committee Review Board and complied with federally-mandated animal care and use guidelines (Permit number: ACUC #12–0405). All efforts were made to minimize animal suffering.

*Wheel running protocol*: Mice were randomized into 2 groups; exercise (EX, n = 10) and exercise + stressed (EXS, n = 10). Mice were singularly caged and provided with an in-cage running wheel (4.5” Mouse Wheel, Respironics, Bend, OR, USA). All mice (n = 20) were given free access to running wheels over a 4-week acclimation period to determine running characteristics of each mouse and to ensure that our randomization was effective in terms running time and distance. We determined during the acclimatization period that approximately only one percent of wheel activity occurred during the daytime. EXS mice received exposure to UCSM stress 7 hours per day (8:00 a.m.-3:00 p.m.), 5 days a week for 8 consecutive weeks. Data from the four-week acclimation period confirmed good randomization.

*Stress*: We used a modified UCMS protocol [8] with varying stressors that included, bath (no bedding with approximately 2 cm of water), damp bedding (enough water added to saturate all the bedding), no bedding (all bedding removed to expose mice to plastic bottom of cage), bedding change, cage tilt (cage with no bedding with one end propped up to create a 45° angle, forces mice to remain in corner of cage), altered light cycles (varying durations of altering the lights on or off in the room), and social stress (mice were shifted to their neighbors cage to expose them to a new environment and scents, a holding cage was used so there was no interaction amongst the mice). The 7 hours of stress, given from approximately 8:00 a.m.-3:00p.m., consisted of short, 1–3.5 hours changing blocks of stressors. The ordering and schedule of stressors were changed each day to ensure that mice would not habituate to the stress routine. An example of the weekly stress protocol can be found in Table 1 (this schedule changed weekly). During the weekday (Monday through Friday), all mice were given access to running wheels from approximately 3:30 p.m. to the following morning (7:30 a.m.). At 7:30 a.m. overnight data for time and distance was collected using a odometer (Strada CC-RD 100N CatEye America, Inc. Boulder CO) with a sensor attached to the running wheel. For stress exposure, the EXS group were placed in new cages identical to their main cage, but without the running wheel, and transported to the lab for the 7 hour stress protocol each weekday. At the same time (7:30 a.m.– 3:30 p.m.), the EX (no stress) group had their running wheels locked so they could not exercise while the EXS underwent stress exposure. After the stress exposure, the EXS group was returned to their main cage and both EXS and EX groups were allowed to use the running wheels in their cage. Data from one EX mouse was excluded from the study due to a loss of equipment from the animal chewing on wires.

**Table 1:** 

Monday (Weigh Animals)	Tuesday	Wednesday	Thursday	Friday
Cage Tilt (2.5 hours)	No Bedding (3.5 hours)	Cage Tilt (2.5 hours)	Bedding (Change every 30 mins for 3 hours)	Damp Bedding (2 hours)
Damp Bedding (2.5 hours)	Damp Bedding (3.5 hours)	Bath (30 mins)	Bath (1 hour)	No Bedding (2 hours)
Bath (30 mins)	Social Stress (last 2 hours)	Light (1 hour)	Social Stress (30 minutes)	Cage Tilt (1 hour)
Bedding Changes (every 30 mins for 1.5 hours)	Light/Dark (Every 15 mins for 3 hours; Start at same time as damp bedding)	Damp Bedding (3 hours; Start at same time as Light)	Light/Dark (Every 30 mins for 3 hours; Start same time as Social Stress)	Light (2 hours; Bath at same time)
		Social Stress (last 60 mins)		

All mice were also given unrestricted access to running wheels during the weekends (a time period of no stress for EXS mice) by leaving running wheels unlocked from 3:30pm Friday until 7:30 am Monday (denoted as EX-WE and EXS-WE). Run time and distance were collected each Monday morning and data was divided by three and averaged for weekend daily amounts. Weekend and weekday data are evaluated separately in the analysis. Mice were weighed at the beginning and the end of every week and this was the only time the EX group was handled during the 8-week protocol. Cages were cleaned at the same time in order to limit handling.

*Indicators of stress*: Previous research has shown that bladders from mice subjected to chronic stress undergo significant morphological change including hypertrophy and enhanced bladder-to-body weight ratios [20]. This index of stress was chosen because it demonstrates a physiological consequence to chronic stress. The bladders of animals were collected immediately following euthanasia. Bladders were vertically bisected, blotted to remove excess fluid and weights recorded. Weights are reported as average bladder weight in milligrams ± SE. Blood samples were collected and immediately centrifuged, serum obtained and flash frozen in liquid N2 and stored at -80C until analysis. Cortisol was analyzed using mouse Cortisol Enzyme Immunoassay Kit (#IRAAKT2546, Innovative Research, Inc., Novi, MI, USA). Limit of detection of the assay is 45.4 pg/ml, with intra assay co-efficient of variation (CV) of 14.7% at cortisol concentration ~0.18 ng/ml, and intra assay CV of 10.9% with cortisol concentration at ~0.20 ng/ml.

*Statistics*: Statistical analysis was run using SPSS software. All data is shown as mean ± SE. One-tail unpaired t-test was used for single time point measures for cortisol and bladder mass, other measures were analyzed using a one-way ANOVA with Tukey post hoc to compare between groups. Repeated measures ANOVA with Bonferroni post hoc were used to assess weight, weekly, and weekend wheel activity data.

## Results

**Fig 1:**
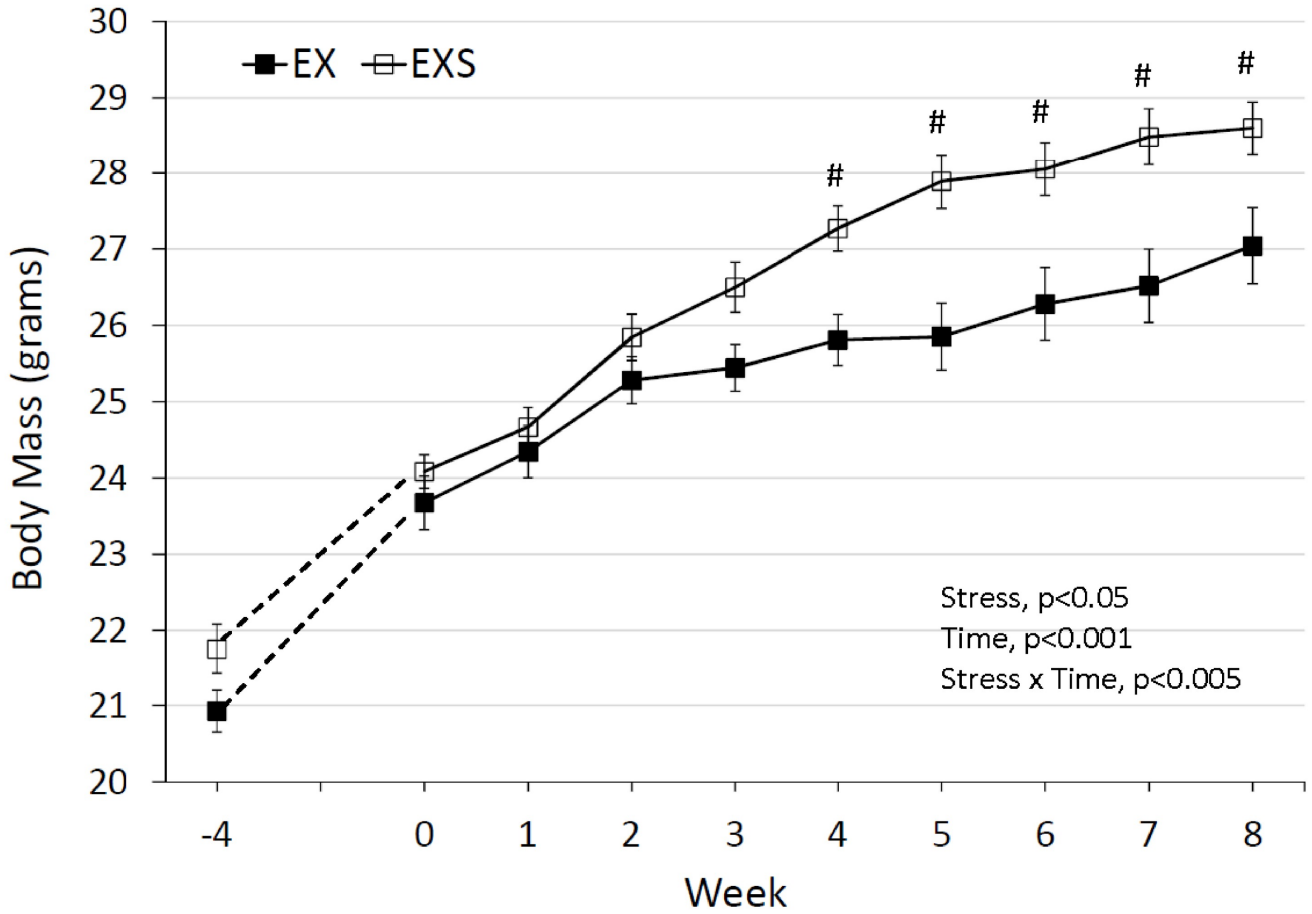
Body mass over the 8 weeks of training in mice subjected to either unpredictable chronic mild stress (EXS) or no stress (EX). Week -4 through 0 represents the pre-intervention period with week 0 as the last weight of pre-training. Repeated measures ANOVA (rmANOVA), EXS increased weight significantly more than EX (interaction main effect, p<0.005); # signifies Fisher’s PLSD p<0.05 compared to EX.

**Fig 2:**
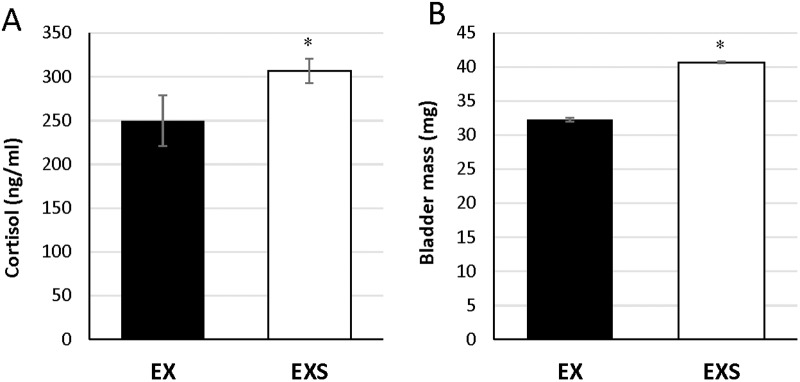
Serum cortisol levels (A) and bladder mass (B) in 8-week non-UCMS exercise trained (EX) and UCMS exercise trained (EXS) mice. Both variables demonstrate greater stress in UCMS treated compared to non-UCMS treated mice. * signifies p<0.05 for unpaired Students T-test.

There was no difference in body mass between EX and EXS prior to stress, as seen in weeks -4 through 0 (start of treatment) in Fig 1, however the increase in body mass was greater in EXS compared to EX (rANOVA df = 1; F-value 8.07; p<0.01, Fig 1). At the end of the 8-week stress period, serum cortisol levels and bladder mass were significantly greater in stressed versus non-stressed mice, respectively (p<0.05, Fig 2). These findings are consistent with the effects of chronic stress [20].

**Fig 3:**
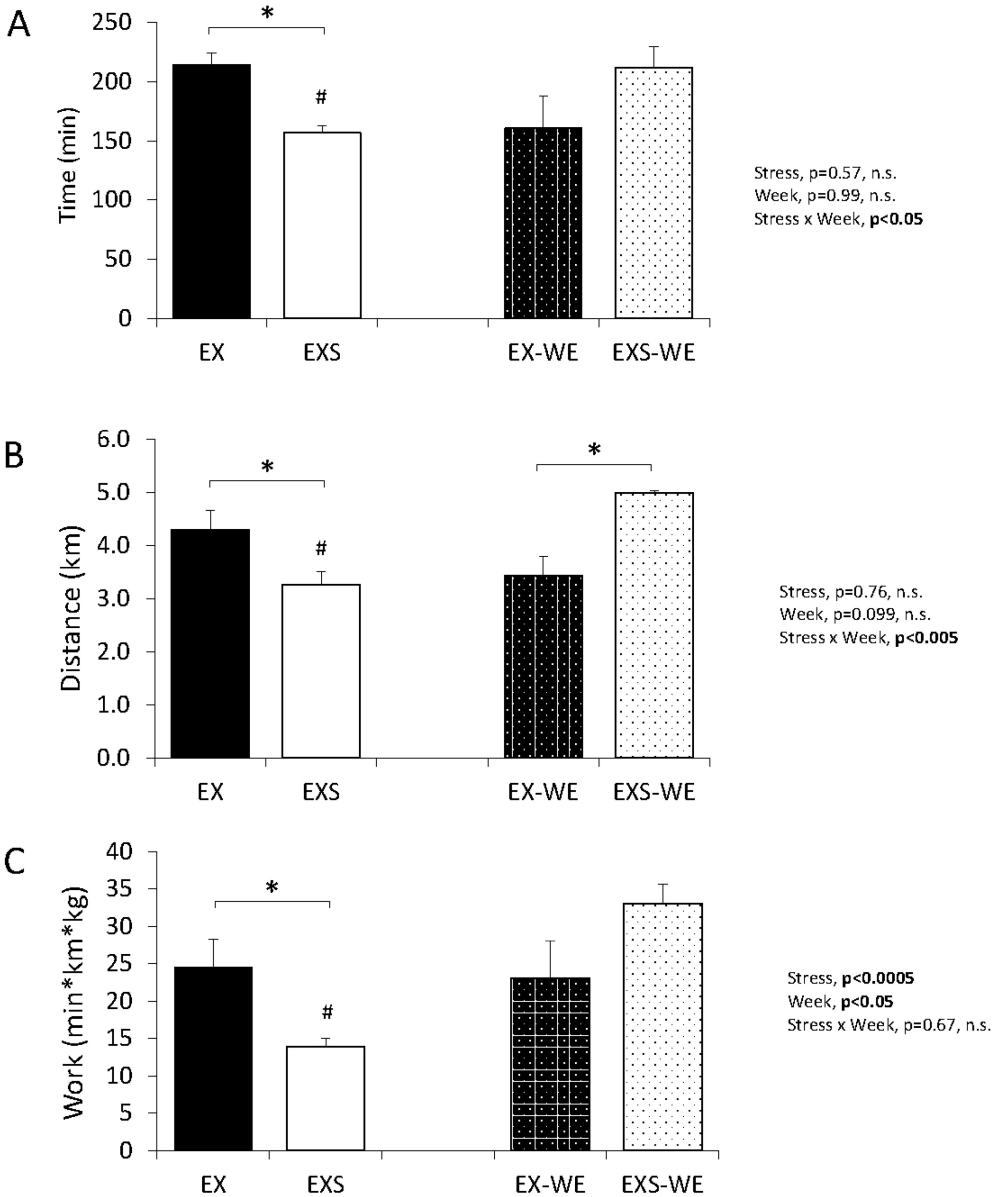
Weekday (EX and EXS) and weekend (EX-WE and EXS-WE) values for the total daily averages of (A) run-time, (B) distance, and (C) energy expenditure, i.e. work (min*km*kg). Two-way ANOVA (stress x time of week), post hoc testing using Fisher’s PLSD, * p<0.05, # denotes difference between weekday and weekend within the same stress group (i.e. EX vs EX-WE or EXS v EXS-WE) p<0.05.

**Fig 4:**
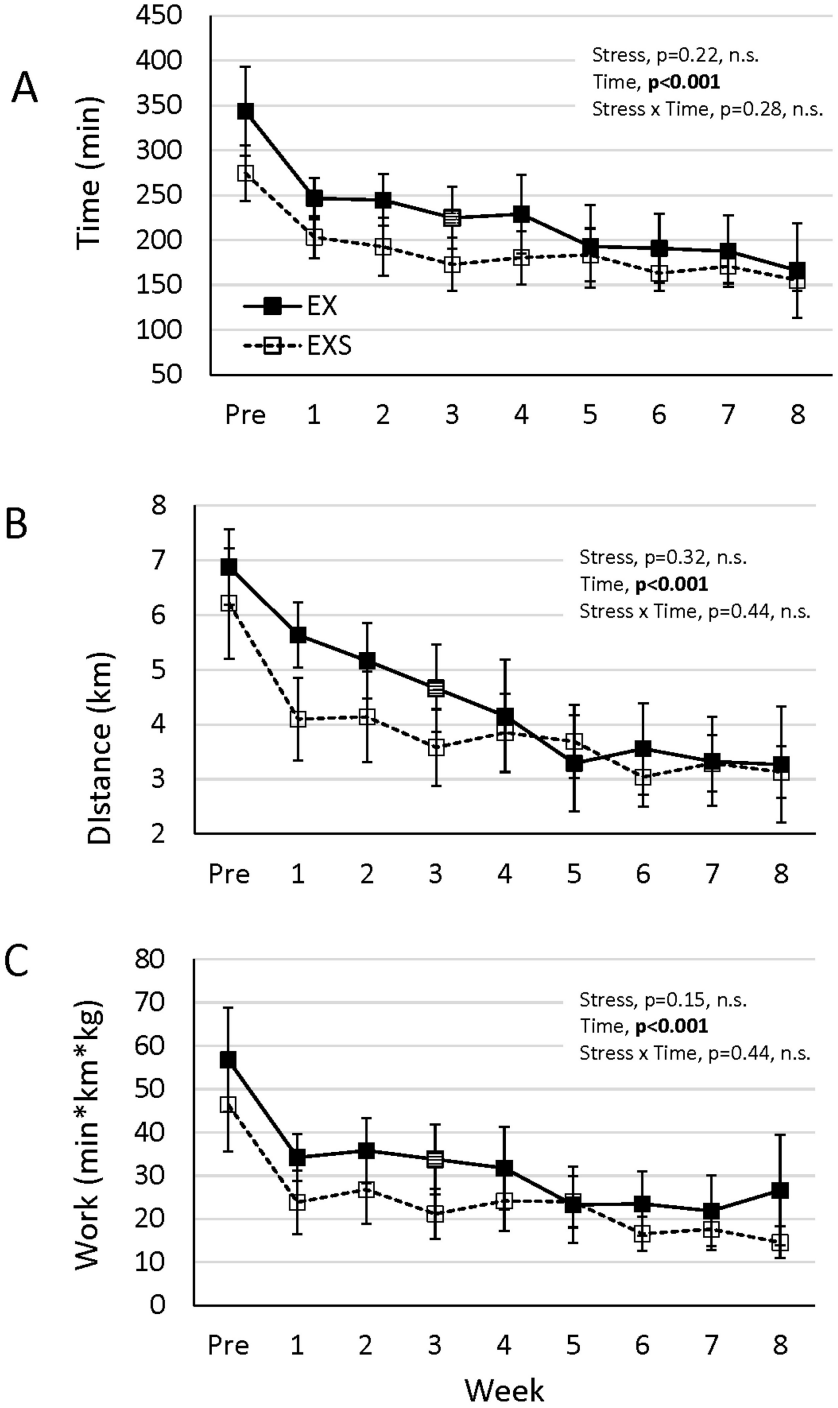
Average weekday wheel activity over the 8-week study for EX and EXS for (A) run time, (B) run distance, and (C) energy expenditure (i.e. work). Data for EX group in week 3 (box with horizontal hash marks) have been imputed based on the average between week two and four values from EX group. Repeated measures ANOVA

The average time spent running each weekday was significantly lower in the EXS compared EX, (Fig 3A, p<0.05). Likewise, the average distance run each weekday was significantly lower in EXS compared to EX (Fig 3B, p<0.05). The greatest magnitude of change in running time and distance in EXS mice (i.e. delta between any two adjacent time point measure) occurred at week 1 (Fig 4), after which the difference remained relatively consistent between the groups. Due to technical errors occurring during week three of the experimental protocol, running wheel data for only the EX group was unavailable, and therefore not included in analysis. However, in Fig 4, for graphical representation only, we have imputed an estimated group mean value for week 3 by taking the average between the week immediately before and after this point (i.e. week 2 and 4, respectively). It is should be noted that statistical analysis outcomes was not significantly altered with or without the imputed values for EX group in week 3. It is evident even by causal observation that data at this one-time point does not change the overall interpretation of our results and therefore does not affect or alter the outcome of the findings.

On the weekend, distance (p<0.001) and time (p<0.05) spent running was higher in EXS-WE versus EXS mice (Fig 3A and 3B). Surprisingly, we also found that distance run over the weekend was significantly higher in EXS-WE versus EX-WE (p<0.001, Fig 3A).

**Fig 5:**
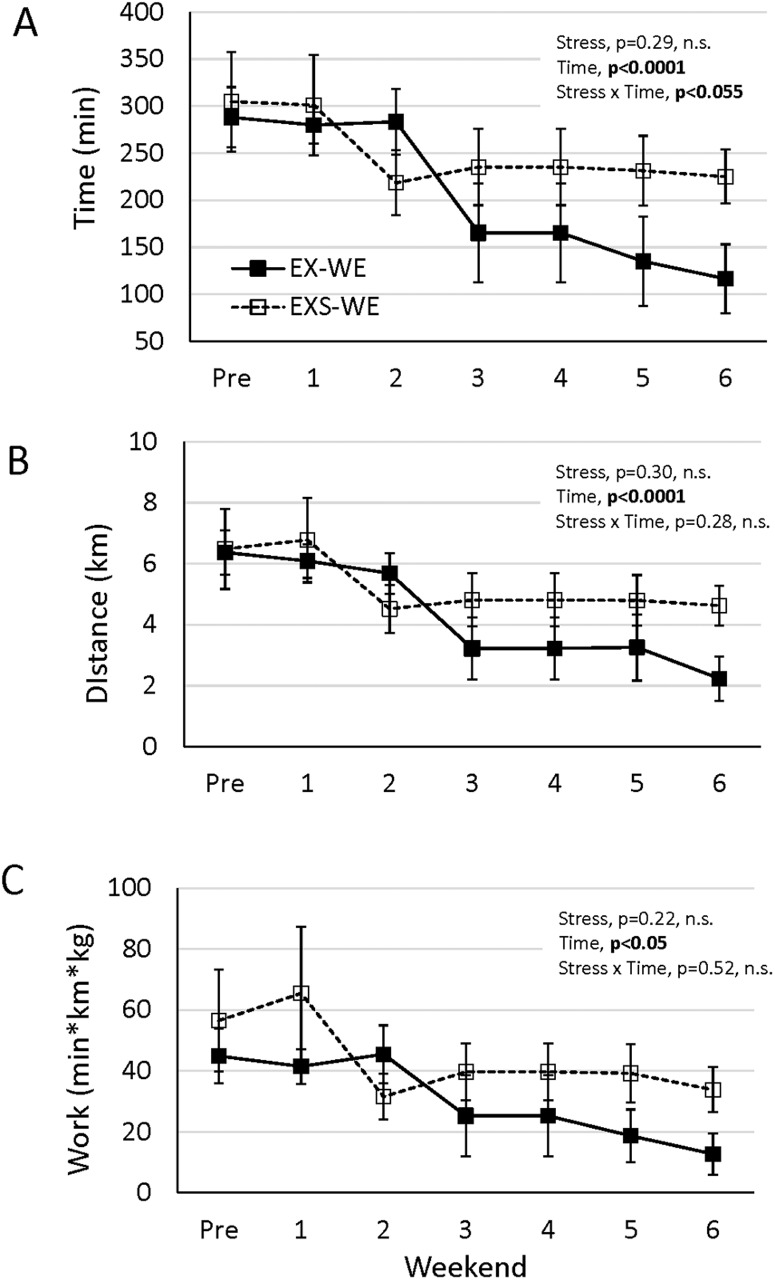
Average weekend wheel activity over the 8-week study for (A) run time, (B) distance, and (C) expenditure. Significant group by time interaction, and significant group difference (p<0.05) are represented in each graph.

Because body weight was different between groups, energy expenditure, defined as total runtime*distance*body mass, was determined for each mouse. EXS-WE had significantly higher expenditure compared to EXS (p<0.01, Fig 3C). Despite the smaller body weight, EX expenditure was significantly higher than EXS (p<0.05, Fig 3C). There was a group by time interaction (p<0.05) for run time on the weekends. It appeared that EXS-WE maintained time spent on the running wheels more so than EX-WE (Fig 5).

## Discussion

To our knowledge, this is the first study to examine the effects of a chronic stress model on long-term wheel running activity in mice and during periods of acute removal of stress. The primary findings are that daily stress reduced voluntary wheel running behavior, and that this effect was most pronounced during the first week, after which the difference between stressed (EXS) and non-stressed (EX) mice remained similar for the duration of the study. Second, when stress was not implemented during the weekend, EXS mice rebounded back to equal or higher levels of activity compared to EX mice.

The pre-intervention activity did not differ between the EXS and EX for any measure assessed, which indicates good randomization and no inherent baseline performance bias in either EX or EXS groups. We found that both EX and EXS mice showed an overall decline in voluntary wheel running activity over the course of the study (Fig 4). This finding is consistent with other murine studies [21,22] showing a decline in wheel running activity starting between 8–10 weeks of age, which is similar to the age of our mice at the beginning of the 8-week stress period. Indeed, Tou and Wade [22] reported a significant negative correlation between body weight and exercise activity in several strains of mice.

Our study also shows the imposition of stress further reduced spontaneous wheel running activity, as observed by less time (about 1 hour less per day) and distance performed by EXS compared to EX mice, which is also consistent with prior evidence of decreased activity after chronic stress [23]. However, in contrast to the prior study, because we assessed multiple time points in our study, we are able to show the greatest decline associated with the imposition of stress occurred within the first week, after which the magnitude of the decline remained consistent between EXS and EX mice. At the start of the UCMS protocol the EXS group wheel activity dropped sharply (i.e. overnight) compared to EX, but thereafter EXS and EX declined at a similar rate over the remaining 7 weeks. The etiology for the immediate drop in overnight wheel activity is not yet known, but may be due to the rapid onset of depressive-like symptoms with UCMS. Mice which have been bred for high voluntary wheel-running demonstrate an exaggerated HPA axis and significantly high glucocorticoid level [6], markers often associated with chronic stress. However, when these mice undergo an acute stressful event they have been reported to exhibit depressive-like behavior and reduced physical activity [7]. Evidence of stress in our mice exposed to 8 weeks of UCMS comes from significantly greater bladder mass and elevated basal serum levels of cortisol when compared with non-stressed animals. We examined changes to the bladder because previous research has shown that bladders from mice subjected to chronic stress undergo significant morphological change including hypertrophy and enhanced bladder-to-body weight ratios [24]. However, changes in bladder mass in Balb/c mice, which are known to be susceptible to UCMS [25,26], have not been previously reported. Our data are consistent with the finding that chronic stress results in elevated bladder mass. We also measured circulating cortisol as an additional indicator of stress and found it to be significantly elevated in the stress mice. Although corticosterone is the primary glucocorticoid in mice and would have served as a better stress indicator, plasma values of cortisol have previously been shown to correlate strongly with corticosterone in mice, throughout the day and after episodes of both acute and chronic stress [27]. There is also general agreement that numerous blood samples should be obtained (over a period of days) to get the best estimates of mean basal glucocorticoid levels and/or several times a day for diurnal slopes [28]. Although our cortisol data are obtained from a single blood sample, which maybe a limitation in our design, they were all taken at the same time of day. We would also be concerned that multiple blood samples taken through the time course of the study would have introduced additional stress to both groups and therefore complicated the interpretation of the data in the non-stressed animals. Nevertheless, collectively our findings are consistent with numerous behavioral studies examining the effect of UCMS exposure in connection with depression-like symptoms in rodents [17,29–33]. We are unable to determine from our results if a depressive-like response was occurring in our mice following five days of UCMS and if this led to the significant decrease in voluntary wheel activity.

Our weekend data (when stress was not implemented) showed an immediate rebound of wheel activity in the EXS mice (i.e. as seen in EXS-WE data in Fig 3). Our goal was to use a schedule of stress and running wheel monitoring that mirrors many exercise training studies with mice; most prolonged training studies use 4–5 day exercise training protocols. The observation of rapid reversal of reduced spontaneous wheel running behavior supports the idea that the negative affect of chronic stress on spontaneous physical activity in rodents may only be transient and temporally linked to the onset of the stress period. While a similar age-related decline in spontaneous wheel running was observed in both groups, the EXS-WE wheel activity was typically greater than EX-WE which may reflect a compensation for the decrease in activity during the week. Our data are supported by similar findings in another study showing a partial rebound in voluntary wheel activity in the weeks following 4 weeks of stress [30]. Malisch et al. recently showed that mice exposed to a single 40-minute restraint stress increased activity the next day during the light phase, a period of typical inactivity. [34] While it is possible that our stressed mice ran more only during Saturday mornings we feel this unlikely. Our stress protocol was for seven hours a day for five straight day, much different from a single 40-minute restraint stressor. Mice were mostly likely still under the influence of stress during the early weekend period because of the volume used throughout the week. In addition, our mice were not restrained but allowed to move freely in their cages during the stress. Finally, we determined during the acclimatization period that almost all of the wheel activity occurred during night in our mice. We believe the compensation would have occurred throughout the weekend, not just in a single time period. However, we are unable to know this for sure and it is a limitation in our study design.

It should be noted that weight gain in response to chronic stress is not a universal finding. Li et al., reported a lower body weight in mice exposed to chronic mild stress as compared with controls [35]. While UCMS is intended to produce only mild stress, it involves a series of unpredictable social and environmental changes that rodents will have difficulty adapting to and therefore likely produce a more constant and continued level of stress compared to any single stress stimulus. Our results showed that the UCMS protocol can alter the extent to which mice exercise which could have had an effect on weight gain. Because we did not measure food consumption in our study, we cannot be certain if the weight gain in EXS mice was due principally to reduce activity or a potential increase in food intake, or some combination of both. However, studies involving reoccurring stress in both mice [36] and rats [37,38] typically show a decrease (and not increase) in food intake, therefore it seems unlikely that greater food intake was responsible for the weight gain in EXS mice. On the other hand, if reduced food consumption was present in our EXS mice, this would have only attenuated the significant gains in body mass observed in EXS mice, and likely further exacerbated the effects of spontaneous activity we observed. This is an issue that will need to be addressed in future studies using the UCMS and exercise.

Another potential reason for the loss in running wheel activity may be due to changes that occur in neural function due to chronic stress. Numerous changes in brain function have been reported in neural and behavioral experiments using the UCMS protocol [8,11,32,39–44]. Several studies have found a decrease in brain neuron signaling, whether through decreases in receptors, loss of plasticity, or increase in apoptotic signaling [33,40–42,44–47]. These experiments reported decreases in neural function following approximately 4 weeks of stress and it is possible this could also affect the willingness to exercise. However, our data does not directly support this idea because the primary decrease in wheel running occurred during week one of the stress protocol and the rebound seen during the weekend was mostly consistent throughout the study.

In summary, repeated daily stress negatively influenced wheel running activity in mice over 8 weeks. However, beyond the decline in voluntary exercise observed during the first week, the reduction in voluntary exercise behavior between stressed and non-stressed mice remained the same during the subsequent 7 weeks of stress. Wheel activity greatly increased on weekends (when the mice were free of the weekday stress), which supports the notion that daily stress has only limited effect on spontaneous physical activity in rodents. Future studies will need to identify the potential neural changes that occur in animals exposed to chronic stress to unravel the mechanism(s) related to chronic stress that reduce spontaneous physical activity.

## Supporting information

S1 File Bryner Minimal DataSet for PLOSone journal. (XLSX)
